# Biological Potential and Essential Oil Profile of Two Wild Apiaceae Species from Algeria (*Daucus carota* L. and *Foeniculum vulgare* Mill.): Larvicidal and Antibacterial Effects

**DOI:** 10.3390/molecules29194614

**Published:** 2024-09-28

**Authors:** Aicha Khemili, Djamel Bensizerara, Haroun Chenchouni, Rachid Chaibi, Nadjwa Aissani, Desiye Tesfaye Tegegne, El-Sayed R. El-Sayed, Antoni Szumny

**Affiliations:** 1 Department of Molecular and Cellular Biology, Faculty of Nature and Life Sciences, University of Abbes Laghrour, Khenchela 40000, Algeria; 2 Laboratory of Biotechnology, Water, Environment and Health (LBWEH), University of Abbes Laghrour, Khenchela 40000, Algeria; 3 Department of Agronomy, Faculty of Nature and Life Sciences, University of Abbes Laghrour, Khenchela 40000, Algeria; 4 Laboratory of Algerian Forests and Climate Change (LAFCC), Higher National School of Forests, Khenchela 40000, Algeria; 5 Laboratory of Natural Resources and Management of Sensitive Environments (RNAMS), University of Oum-El-Bouaghi, Oum-El-Bouaghi 04000, Algeria; 6 Laboratory of Biological and Agricultural Sciences (LBAS), University of Amar Telidji, Laghouat 03000, Algeria; 7 Department of Pharmacology and Toxicology, Faculty of Veterinary Medicine, Wrocław University of Environmental and Life Sciences, Norwida 31, 50-375 Wrocław, Poland; 8 Animal Biotechnology Research Program, National Agricultural Biotechnology Research Center, Ethiopian Institute of Agricultural Research, Holeta P.O. Box 249, Ethiopia; 9 Department of Food Chemistry and Biocatalysis, Faculty of Biotechnology and Food Science, Wrocław University of Environmental and Life Sciences, Norwida 25, 50-375 Wrocław, Poland; 10 Plant Research Department, Nuclear Research Center, Egyptian Atomic Energy Authority, Cairo, Egypt

**Keywords:** infectious diseases, essential oil, Apiaceae, GC/MS, bioinsecticides, antibacterial efficacy

## Abstract

Infectious diseases, including vector-borne and antibiotic-resistant infections, present significant global health challenges, necessitating the exploration of natural alternatives for disease control. In this study, we investigated the essential oil (EO) profile as well as larvicidal and antibacterial properties of two wild Apiaceae species used in Algeria: *Daucus carota* L. (DCEO) and *Foeniculum vulgare* Mill. (FVEO). EO was extracted from the aerial parts by steam distillation and analyzed using Gas Chromatography Mass Spectrometry (GC/MS). Major constituents identified in DCEO were geranyl acetate (50.07%) and elemicin (10.77%), while FVEO contained estragole (24.93%), fenchone (20.20%), and α-phellandrene (17.96%). Both EOs were highly effective towards *Culex pipiens* larvae, with low LC_50_ values of 30.6 ± 1.06 ppm for DCEO and 34.7 ± 1.06 ppm for FVEO, indicating their potential as bioinsecticides due to their bioactivity and bioavailability. Additionally, the two Eos demonstrated moderate antibacterial efficacy against gram-positive bacteria, *Staphylococcus aureus* ATCC 25923 and *Staphylococcus aureus* MRSA ATCC 43300, respectively, with DCEO showing MIC values of 10 and 20 mg/mL, respectively, and FVEO exhibiting MIC values > 20 mg/mL. However, both EOs showed limited effectiveness against gram-negative bacteria, *Escherichia coli* ATCC 25922 and *Klebsiella pneumonia* ATCC 700603. These results highlight the potential applications of DCEO and FVEO as natural bioinsecticides and antibacterial agents, offering promising avenues for further research and development in pest control and food preservation.

## 1. Introduction

The infectious diseases cause acute damage to hosts through direct pathogen invasion, prompting international health organizations to warn about their re-emergence [1]. These diseases, caused by various bacteria, fungi, viruses, and parasites, are among the leading causes of mortality and morbidity globally [2]. Despite significant progress in diagnosis, prevention, treatment facilities, and widespread vaccination across many regions, infectious diseases continue to pose a substantial worldwide threat [3]. Bacterial infections, in particular, represent a significant problem for humanity, with antimicrobial resistance (AMR) becoming an increasingly urgent concern [4]. According to the World Health Organization (WHO) African Region [5], AMR poses a serious and growing threat to public health in Algeria. In 2023, it was reported that *Klebsiella pneumonia* and *Escherichia coli* contributed to over 100,000 deaths each due to AMR. The Algerian Antibiotic Resistance Network (AARN) [6] reported that among 19 bacterial strains isolated from blood cultures in 2020, *K. pneumonia* (23.98%), *Staphylococcus aureus* (16.94%), *Acinetobacter Baumannii* (11.8%), and *E. coli* (9.7%) were ranked as the four leading pathogens. As conventional treatments become less effective due to rising drug resistance, the reliance on herbal medicine grows. Consequently, it is essential for healthcare practitioners to be knowledgeable about herbal remedies [4].

Mosquitoes, particularly those of the Culicidae family, are also highly efficient vectors of infectious diseases, affecting both human and animal health. In North Africa, species within the Culicidae family have historically been linked to severe epidemics. Although known for their painful bites, these insects are the primary vectors of numerous parasitic diseases, especially those transmitted by the *Culex* genus, notably *Culex pipiens* Linnaeus, 1758 [1]. Mosquito control in Algeria primarily relies on the application of synthetic chemical pesticides, such as larvicides and adult repellents [7]. However, the extensive use of these insecticides has led to resistance in *Culex* populations. With increasing mosquitocidal resistance and the limited success of new insecticidal agents in biocontrol programs, there is a pressing need to explore new mosquitocidal agents, such as botanical mosquitocides [1,7].

Moreover, considering the “One World—One Health” approach, it is essential to develop innovative strategies that address the emergence of infections and reduce AMR and mosquitocidal resistance by leveraging the safe potential of essential oils (EOs) [8]. The Apiaceae family, also known as the parsley or carrot family, is one of the largest and most recognized families of Angiosperms, with a wide distribution worldwide [9,10], specifically within the Mediterranean region [11]. Apiaceae plants are recognized for their distinctive pungent aroma, arising from EOs, which find diverse applications in the culinary, medicinal, and personal care sectors [12]. These plants are known for efficiently producing EOs with important chemical diversity [13,14]. EOs extracted from Apiaceae plants are used in cosmetic products for flavoring and fragrance, and they exhibit a wide range of effects, including anti-inflammatory, antimicrobial, antioxidant, antibiotic, antidiabetic, anticarcinogenic, diuretic, cardioprotective, antihyperglycemic, hypolipidemic, and antitumor properties [9,10,13]. In addition to these biological activities, their potential as bioinsecticides is very promising, which makes them a safe and effective eco-friendly alternative [13].

Wild carrot (*Daucus carota* L.) and wild fennel (*Foeniculum vulgare* Mill.) are the most prevalent and renowned plants in the Apiaceae family [10] due to their potent nutritional characteristics [15]. In North Africa, they thrive spontaneously along hillsides, mountainous regions, and in rocky and sandy meadows [16]. Wild plants face greater natural stresses than cultivated plants, leading to increased release of secondary metabolites [10]. Carrot and fennel have been extensively used as natural remedies and flavoring herbs due to their beneficial secondary metabolites, especially volatile components [15]. *D. carota* EO (DCEO) and *F. vulgare* EO (FVEO) are attracting considerable scientific interest due to their numerous pharmacological activities [15].

Therefore, in this article, we aimed to conduct a comprehensive and comparative analysis of the EO profiles of two well-known wild plants, *D. carota* and *F. vulgare*, from northwestern Algeria. Additionally, we explored the biological potential of these plants by (i) assessing their larvicidal effect against *C. pipiens* larvae and (ii) evaluating their antibacterial activity. While DCEO and FVEO have been previously examined for their antibacterial and larvicidal activities, it is crucial to recognize that the results of such studies are not universally consistent due to the inherent variability in EO composition and biological effects, which are influenced by the geographical and environmental conditions of plant growth. EOs are secondary metabolites, whose production and chemical profiles are highly sensitive to local environmental factors such as climate, soil properties, altitude, and habitat characteristics [17,18]. These factors contribute to the distinctiveness of EOs even within the same species, leading to variations in yield, chemical composition, and consequently biological activities.

## 2. Results and Discussion

### 2.1. Extraction Yield

Wild carrot and fennel exhibit sequential flowering and maturation of their inflorescences, making the harvest period crucial for achieving a high yield of EOs [19]. In this study, the extraction yields of DCEO and FVEO from the aerial parts were determined to be 0.80% and 0.85%, respectively. The DCEO extraction yield closely aligned with the results previously reported by Servi et al. in Turkey [20]. In western Algeria, the DCEO yield was found to be 0.52% [21] and 1.52% [22], while in eastern Algeria, it was 0.61% [16]. The DCEO yield was reported to be 2.1% from leaves in northern Algeria [23], and in Portugal, the DCEO yield from umbels was 0.9% [24]. However, the FVEO yield in this study was consistent with that of Hamada et al., who documented a yield of 0.89% in the southeastern region of Algeria [25]. A study from northeastern Algeria indicated a good FVEO yield of 1.40% [26]. The highest FVEO yield reported in the literature was obtained from Egyptian FVEO extracted from aerial parts, at 2.5% [27]. In contrast, FVEO from aerial parts in thirteen different Italian regions ranged from 0.04 to 0.38% [28], while in Portugal it was reported as 0.29% [29], in Tajikistan as 0.5% [30], and in Turkey as 0.77% [20]. As evident from these diverse results, significant variation in EO production has been documented, influenced by numerous elements, namely the variety of the plant, origin of the selected plant, plant part, growth phase, extraction techniques, duration of extraction, and harvest period. EOs can be found in all parts of the plant, albeit in varying compositions and concentrations [20,31,32].

### 2.2. Chemical Profile of EOs

The chemical analysis of EOs was carried out by GC/MS analysis, revealing 84 compounds in DCEO, which accounted for 95.97% of volatile compounds, and 42 were identified in FVEO, representing 98.84% of the volatile compounds (Table 1).

**Figure 1:**
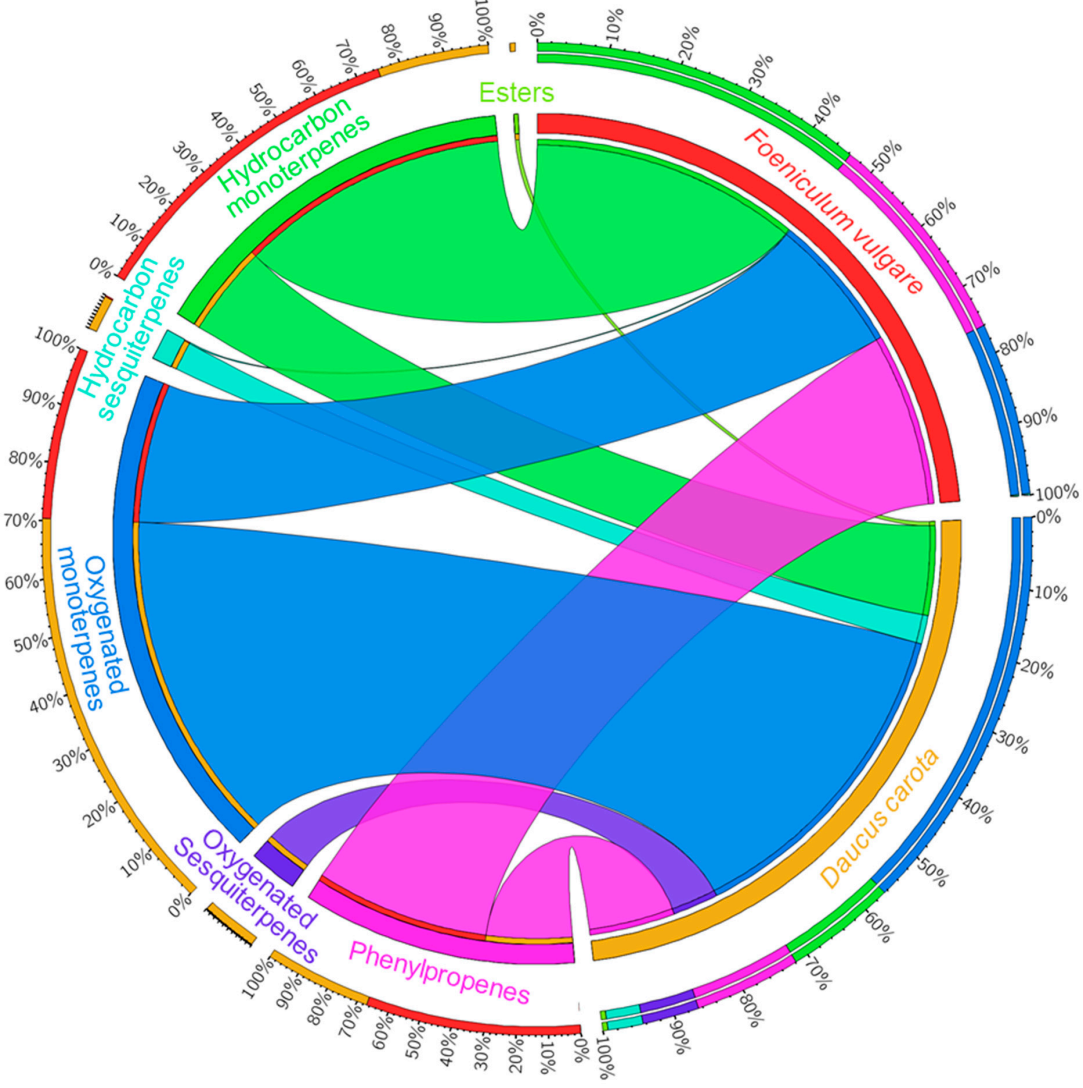
Percentage of chemical families identified in DCEO and FVEO.

**Figure 2:**
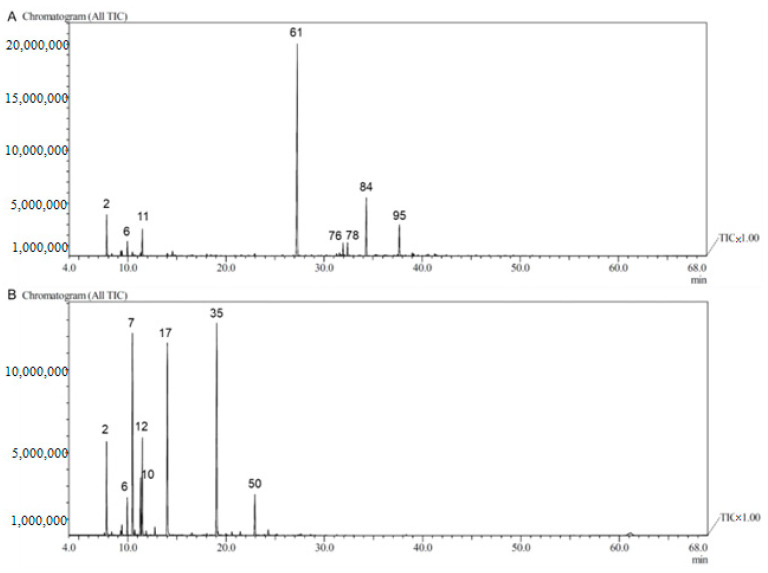
The TIC chromatogram of DCEO (**A**) and FVEO (**B**); numbers associated with chromatogram peaks are represented in Table 1.

Compounds in DCEO were categorized as oxygenated monoterpenes (54.17%), hydrocarbon monoterpenes (14.76%), phenylpropenes (13.62%), oxygenated sesquiterpenes (7.56%), hydrocarbon sesquiterpenes (4.69%), and others (1.18%) (Figure 1). The major family was the oxygenated monoterpenes, with a dominant presence of geranyl acetate (50.07%). The phenylpropene compounds, elemicin and methylisoeugenol, were abundant with 10.77% and 2.21%, respectively. Himachalol (5.92%) was the major oxygenated sesquiterpene. The hydrocarbon monoterpenes were abundant with α-pinene (5.44%), limonene (4.23%), and β-myrcene (2.06%). Overall, β-bisabolene (2.40%) was the predominant component among the hydrocarbon sesquiterpenes (Figure 2A, Table 1). 

In many investigations, hydrocarbon monoterpenes were identified as the predominant components in DCEO from different aerial parts [16,21,22,24,33,34,35], with α-pinene, sabinene, and limonene being the most dominant. DCEO was classified as a sabinene chemotype in Lithuania [36] and Serbia [37,38]. However, the presence of oxygenated monoterpenes was significantly lower in these studies compared to the current study, where geranyl acetate (50.07%) was the main compound. Our results closely align with those of a study conducted by Ksouri et al. in northern Algeria, where oxygenated monoterpenes (66.08%) were the dominant group, represented by geranyl acetate (52.45%) [23]. This compound was also identified as the major component in DCEO from two Portuguese regions, whereas it was absent from the ones in Italy [24,39]. Sesquiterpenes were the dominant group in DCEO from Tunisia [40,41], Turkey [20], and Montenegro [19]. Furthermore, there were significant quantitative differences in the principal compounds of DCEO, particularly in the phenylpropanoid content. In France, methylisoeugenol (21.8%) and elemicin (16.3%) were the major compounds [42]. Elemicin (18.1%) was also the major compound in Turkish DCEO [20]. Few studies, including this one, have not reported the presence of carotol, daucene, and daucol, the sesquiterpenes typically found in the chemical profile of DCEO [19,21,22,33,34], although they were present in small amounts in eastern Algeria [16].

The significant class represented in FVEO was hydrocarbon monoterpenes (45.71%), dominated by α-phellandrene (17.96%), β-phellandrene (9.14%), α-pinene (7.36%), *p*-cymene (4.88%), and β-myrcene (2.88%). The phenylpropene group (29.41%) was represented by estragole (24.93%) and anethole (4.48%). Fenchone (20.20%) was the major component of oxygenated monoterpenes (23.53%). There was a weak presence of hydrocarbon sesquiterpenes (0.18%) and an absence of oxygenated sesquiterpenes (Figure 1, Figure 2B, Table 1). Thus, the major components were estragole (24.93%), fenchone (20.20%), and α-phellandrene (17.96%). Estragole also emerged as the predominant component in some studies, ranging from 20.25% to 89%, observed in both aerial parts and seeds [20,31,43,44,45]. Estragole and fenchone were present in moderate amounts in various investigations [46,47,48,49,50,51]. Anethole, or its isomer, was the main compound found in many studies across different parts of the plant [29,32,38,45,46,47,48,49,50,51]. Other studies in Algeria produced distinctly different results about FVEO. α-phellandrene (29.44%) was the primary compound in aerial parts [25]. Fenchone (83.63%) was the predominant compound in seeds [52]. Camphor, fenchone, and *o*-cymene were the major compounds in aerial parts [26]. α-thujene and carvone were the major substances in leaves and flowers, respectively [25]. 

Various factors, especially environmental factors and climatic conditions, can significantly alter the biosynthesis of volatile compounds present in EOs, leading to modifications in the chemical composition. Consequently, different chemotypes often exist within the same species. These differences affect the biological activities of EOs, which explains the widely varying results reported in several studies [12].

**Figure 3:**
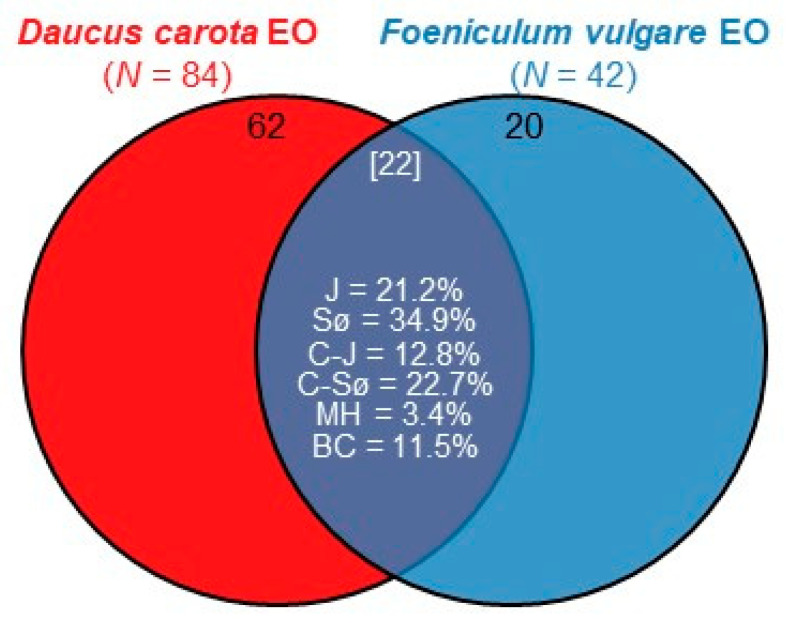
Venn diagram displaying the distribution of chemical components detected in DCEO and FVEO. Figures in black are number of exclusive components in plant species, whereas the white number between square brackets represents the number of shared components among these species. Similarity statistics (in %) are displayed within the overlapped area of the diagram. (J: the classic Jaccard index, Sø: the classic Sørensen index, C–J: Chao’s abundance-based Jaccard index, C–Sø: Chao’s abundance-based Sørensen index, MH: Morisita–Horn index, BC: Bray–Curtis index).

According to paired *t*-tests, variations in the chemical components between EOs from the two plants did not show a significant difference (*p* < 0.05) for each chemical family. Figure 3 shows that 22 compounds were shared between the two EOs, including hydrocarbon sesquiterpenes, phenylpropenes, and hydrocarbon and oxygenated monoterpenes, with α-pinene (5.44%, 7.36%) and β-myrcene (2.06%, 2.88%) both present in significant concentrations in DCEO and FVEO, respectively. Overall, the similarity of chemical composition among the two EOs was low, with qualitative similarity scores ranging between 21.2% and 34.9%, obtained using Jaccard and Sørensen indices, respectively. The estimates of quantitative similarity were also low; the value of Morisita–Horn index was 3.4%, and Bray–Curtis index was 11.5%. Our study, focusing on wild-growing populations of *D. carota* and *F. vulgare* in Algeria, reveals new insights by highlighting how specific ecological conditions, unique to the region, influence the EO profile and its subsequent biological potential. This regional specificity is crucial, as previous studies often overlook the subtle, but significant, impact of local environmental conditions on EO characteristics. According to Mehalaine and Chenchouni, plants growing in the same geographic area can exhibit substantial differences in metabolic output due to varying soil properties and climatic factors, making the study of EOs context-dependent and continually relevant [17,18].

### 2.3. Larvicidal Activity against Culex pipiens Larvae

Exploring eco-friendly alternatives in botanicals, such as EOs, is an urgent necessity. Numerous EOs have been employed to manage agricultural pests, medically significant viruses, and vectors. They are regarded as safe and environment-friendly bioinsecticides due to their rapid degradation in water and soil, minimal toxicity to mammals, and effectiveness against various insects [53]. Furthermore, their diverse mechanisms and target sites pose challenges for mosquitoes in developing resistance [53]. Mosquito management often focuses on the application of larvicides to control larvae in their breeding habitats, as adulticides typically provide only a temporary reduction in the adult population [48]. At present, bioinsecticides are seldom used in mosquito larval populations [13]. To our knowledge, the larvicidal activity of FVEO and DCEO from aerial parts against *C. pipiens* larvae has not yet been reported in Algeria. 

**Figure 4:**
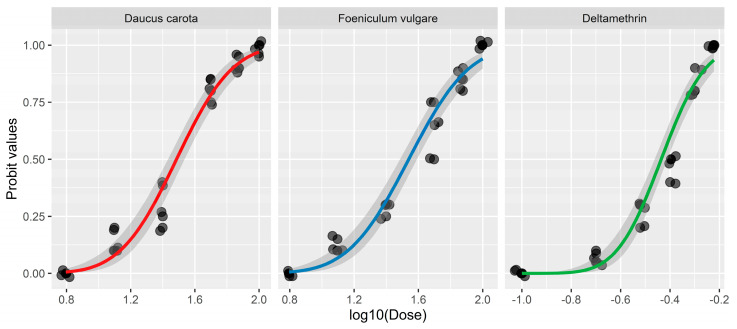
Probit responses of DCEO, FVEO, and the insecticide “Deltamethrin” plotted against *Culex pipiens* larvae mortality.

**Figure 5:**
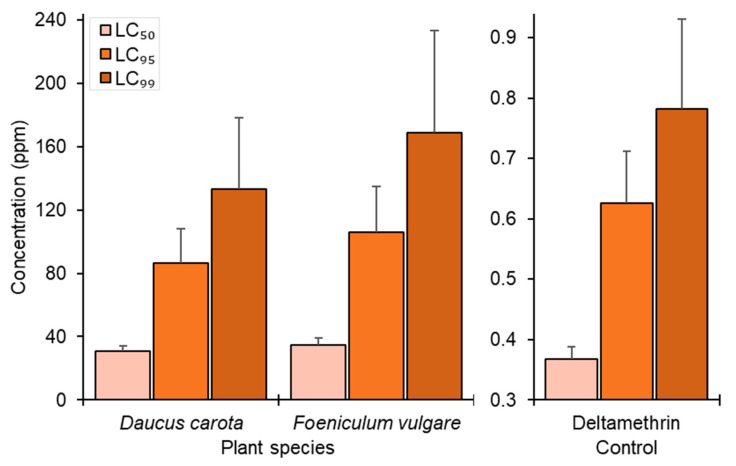
Lethal concentrations LC_50_, LC_95_, and LC_99_ of DCEO, FVEO, and the control “Deltamethrin” against *Culex pipiens* larvae. Vertical error bars represent upper 95% confidence limits.

In the current study, the larvicidal potential retained by DCEO and FVEO from aerial parts was investigated, and the results are presented in Table 2. The EOs were considered strongly effective, showing larvicidal mortality rates against *C. pipiens* of 95–100% at 100 ppm. At 50 ppm, DCEO exhibited a high larvicidal mortality (80% ± 5.00), followed by FVEO (63.33% ± 12.58). These EOs induced larval mortality with a concentration–response relationship (Figure 4). DCEO and FVEO exhibited strong larvicidal effects against *C. pipiens*, showing LC_50_ values below 35 ppm, 30.6 ± 1.06 ppm, and 34.7 ± 1.06 ppm, respectively, and induced morphological deformation (Figure 5, Table 2). 

This indicated that the DCEO and FVEO extracted in our study were significantly more effective against *C. pipiens* larvae compared to DCEO extracted from umbels in Peoria, which had an LC_50_ of 42.9 ppm [53], and FVEO extracted from seeds in Algeria, which had an LC_50_ of 40 ppm [48]. This variation in effectiveness could be attributed to the differing compositions of the oils. The strong effect observed was likely due to the presence of phenylpropanoids, known for their mosquito larvicidal and molluscicidal properties [54]. Additionally, monoterpenes have been approved by the U.S. Food and Drug Administration as safe bio-larvicides [55]. The hydrocarbon monoterpenes, *p*-Cymene, α-pinene, 3-carene, limonene, and myrcene, present in our oils, achieved LC_50_ values of 22.06, 38.01, 47.04, 53.97, and 72.28 ppm, respectively, against *C. pipiens* larvae [56]. However, geranyl acetate, the predominant oxygenated monoterpene in our DCEO, showed low toxicity (LC_50_ = 135.78 ppm) [56]. Overall, our EOs outperformed 12 individually tested monoterpenes [57]. In this context, the insecticidal effectiveness of EOs appears to depend not only on their specific chemical profile and the total amount of major compounds but also on the relative proportions of both the major and minor compounds, which can result in either synergistic or antagonistic effects [58]. Considering the LC_50_ values of DCEO and FVEO, they could be effective natural insecticides in pest management. Their use can assist in controlling *C. pipiens* populations and lowering the risk of the diseases they spread.

Deltamethrin, a synthetic insecticide used in this study, demonstrated 100% larvicidal mortality at 0.6 ppm, with an LC_50_ of 0.367 ± 1.03 ppm. While our findings suggested that DCEO and FVEO exhibited lower toxicity to mosquito larvae than commonly used synthetic insecticides like Deltamethrin, it is crucial to note that synthetic insecticides are roughly a million times more toxic and hazardous to human well-being and biodiversity than EOs. The continuous use of insecticides has led to their increased accumulation in the environment, disrupting the food chain. Humans exposed to these insecticides, whether through occupation or contaminated water sources, experience toxic effects [58].

### 2.4. Antibacterial Activity

The antibacterial potency of DCEO and FVEO against four pathogens is summarized in Table 3. DCEO showed larger inhibition zone diameters (IZDs) against both *S. aureus* (18.67 mm) and *S. aureus* MRSA (24 mm) strains, though it had high minimum inhibitory concentration (MIC) values of 10 and 20 mg/mL, respectively. In contrast, FVEO exhibited moderate IZDs of 10.33 and 10.67 mm, with MIC values higher than 20 mg/mL. Despite the higher MIC values, the larger IZDs suggested that DCEO had more potent antibacterial activity against gram-positive bacteria than FVEO. Both EOs demonstrated weak antibacterial activity against gram-negative bacteria, *E. coli* and *K. pneumonia*. DCEO exhibited IZDs of 7 and 8 mm, respectively, while FVEO showed an IZD of 9.33 mm for both pathogens. These results were comparable to the IZDs reported with GMN10, which were 30 mm for *E. coli* and 25 mm for *K. pneumonia.*

The rise of bacterial infections caused by multi-resistant strains, coupled with the escalating economic burden, has posed a substantial public health problem. Consequently, the importance of EOs with broad-spectrum antimicrobial properties has grown, leading to their use in food packaging and as coatings to safeguard food products [12]. According to Pesavento et al., our antibacterial findings indicated that DCEO exhibited stronger antibacterial properties against gram-positive bacteria than against gram-negative bacteria [59]. However, due to the high MIC values, the antibacterial efficacy against gram-positive bacteria can be considered moderate, which was consistent with previous research findings [22]. In contrast, the antibacterial efficacy against gram-negative bacteria can be considered weak. Several studies have indicated that gram-positive bacteria exhibit greater susceptibility to EOs compared to gram-negative bacteria, which display higher tolerance levels [35]. The observed distinction arises primarily from the presence of the outer membrane that encases the cell walls of gram-negative bacteria. This membrane comprises lipopolysaccharide chains that restrict the diffusion of hydrophobic constituents, such as EOs [24,60]. Additionally, multiple investigations have reported significant antibacterial activity of DCEO against gram-positive bacteria and limited antibacterial activity against gram-negative bacteria [24,35,60,61,62,63]. The DCEOs investigated in these studies were abundant in hydrocarbon monoterpenes (α-pinene and/or sabinene chemotypes) and/or oxygenated monoterpenes (geranyl acetate chemotypes), which emerged as pivotal constituents contributing to the gram-positive antibacterial efficacy [35,60]. Nevertheless, numerous oxygenated monoterpenes showed weak to moderate antibacterial activity against both gram-negative and gram-positive bacteria [64]. However, β-bisabolene chemotypes revealed strong antibacterial activity against *E. coli* [19,41], which highlighted the reduced DCEO activity reported in this study against *E. coli*, given that β-bisabolene was present at only 2.40%. Regarding the FVEO studied, it exhibited moderate activity against gram-positive bacteria and weak activity against gram-negative bacteria, which is consistent with [32,50]. FVEO had greater sensitivity to both gram-negative and gram-positive bacteria, attributed to higher concentrations of volatile compounds, particularly phenylpropenes [20,45,65,66]. No notable antibacterial effect was observed with FVEO from Portugal [29]. Ahmad et al. indicated that the antimicrobial power of FVEO varies depending on the dose [66].

Due to their moderate antibacterial activity against gram-positive bacteria, DCEO and FVEO are effective in extending the shelf life of industrial products by inhibiting the growth of spoilage pathogens and preventing contamination. As a result, these oils hold potential as natural preservatives in both food and cosmetic products [67,68]. Additionally, these oils could be utilized alongside conventional antibiotics in combination therapies to improve treatment outcomes and minimize antibiotic resistance [67]. This study also seeks to promote the utilization of DCEO and FVEO in combination, either with each other or with nanoparticles, as non-chemical approaches. Such combinations may prolong the effectiveness of these volatile compounds and enhance their physicochemical properties.

## 3. Materials and Methods

### 3.1. Plant Material and EO Extraction

Fresh aerial parts of wild *D. carota* and *F. vulgare* plants were harvested in May 2023 (at the start of the flowering stage) from Sidi Medjahed Forest, Tlemcen, Algeria (34°43′45″ N, 01°34′36″ W). The plants were verified by botanist Dr. Azzeddine Zeraib, and the following voucher specimens have been deposited at the herbarium of the University of Abbes Laghrour, Khenchela, Algeria, for future reference: 2023AKDC for *D. carota* and 2023AKFV for *F. vulgare*. The plant material was air dried for 15 days and protected from direct sunlight. 

EO was extracted using a 1.5 h steam distillation method, following the protocol of Petrović et al. [69]. To ensure optimal yield and quality, we employed a shorter distillation time at a temperature of 104 °C and a water vapor pressure of 0.4 bar. After distillation, the EO was collected, dried over anhydrous sodium sulfate, and the yield was calculated. The oil was then stored in a hermetically sealed amber vial and conserved at an obscurity of −18 °C until further evaluation.

### 3.2. GC/MS Characterization of EO

EO composition was analyzed using a Shimadzu GC/MS QP 2020 (Shimadzu, Kyoto, Japan), equipped with a Zebron ZB-5 MSi capillary column (30 m × 0.25 mm × 0.25 µm; Phenomenex, Torrance, CA, USA). GC oven temperature was programmed from 50 °C to 250 °C at a rate of 3.0 °C and kept for 3 min. Helium gas served as the carrier gas, with a linear velocity of 36.3 cm/s and a column flow of 0.93 mL/min, with a split ratio of 50. The MS operational parameters were as follows: interface and ion source temperature, 250 °C; scan mode, 40–450 *m*/*z*, in mode of 3 scans/s. Approximately 20 µL of EO was dissolved in cyclohexane and injected with 1 µL at 250 °C.

For identification of EO components, Kovats retention index (KI) of each compound was calculated using a KI macro [70] by applying retention times (RT) of C8–C24 n-alkanes (Sigma-Aldrich, Steinheim, Germany) as reference compounds. The components were quantified by comparing their experimentally determined KI and spectral mass with library mass spectral databases like NIST 20 (National Institute of Standards and Technology) and FFNSC 3.0 (Mass Spectra of Flavors and Fragrances of Natural and Synthetic Compounds). Quantification of identified compounds was performed through peak area normalization of GC/MS chromatograms against added internal standard. Search filters were set for mass spectra similarity ≥ 80%. Softwares such as GC/MS Postrun analysis version 4.45 (Shimadzu Company, Kyoto, Japan) and AMDIS GC/MS analysis version 2.73 were employed for data analysis.

### 3.3. Larvicidal Effect against Culex pipiens

First instar larvae of *C. pipiens* (Insecta: Diptera: Culicidae) were randomly collected in March 2024 from standing water pools at the Oued Fritis site, Messaad, Algeria (34°18′43.06″ N latitude, 03°36′38.4″ E longitude, with an altitude of 1.28 km). This site is rich with a high larval density of *C. pipiens*. The species were reared in plastic trays (26 × 20 × 8 cm^3^) with tap water and were kept in the lab environment at 26 ± 2 °C and a photo regime of 14:10 h (light/dark). The larval colonies in the aquatic environment were supplied daily with powdered commercial yeast.

Larvicidal toxicity was assessed using the immersion method, following the standard assay recommended by the WHO and Wangrawa et al. [71,72], with minor modifications. An EO stock solution of 10,000 ppm was prepared in dimethyl sulfoxide (DMSO). Twenty larvae at the final third instar and/or the fourth instar were transferred separately into disposable cups of 240 mL containing distilled water. An appropriate volume of various concentrations (6.25–100 ppm) was added to the cups to achieve a final volume of 100 mL. In parallel, Deltamethrin 25 g (*w*/*v*) (DELTAMAC^®^ 2.5 EC), manufactured by The Poison Control Center, Algiers, Algeria., at concentrations ranging from 0.1 to 0.6 ppm, was served as a positive control. DMSO only was served as a negative control. Three replications were conducted for each of the larvicidal bioassays as well as for the control assays. Larval mortalities were registered at 24 h post-contact, and larvae were not provided with any feed during this period. Dead larvae were recognized by their immobility when probed with a fine needle. The mortality % of each assay and concentration was determined by Formula (1) [73]. In this study, a correction was not required as the negative control mortality was 0%.

(1) $$\mathrm{Mortality}\;\%=\frac{number\;of\;killed\;larvae}{number\;of\;exposed\;larvae}\times 100$$

### 3.4. Antibacterial Activity Assay

Two gram-negative bacteria, *E. coli* ATCC 25922 and *K. pneumonia* ATCC 700603, and two gram-positive bacteria, *S. aureus* ATCC 25923 and methicillin-resistant *S. aureus* MRSA ATCC 43300, were used. Pathogenic bacterial strains ATCC were sourced from the Pasteur Institute of Algeria. They were inoculated from frozen stock (−20 °C) onto Mueller Hinton agar (MHA) and incubated at 37 °C for 24 h. The initial screening of the antibacterial assay of pure EO was conducted using the disk diffusion test in triplicate, following the protocol by Rocha et al. [74]. Positive control disc contained the standard antibiotic, Gentamicin 10 µg GMN10 (Bio-Rad, Watford, UK).

Based on the disk diffusion results, two bacteria were chosen to determine the MIC value using the broth microdilution method [75]. Serial double dilutions of EO were prepared in Mueller–Hinton broth (MHB) containing 5% DMSO in sterile 96-well plates, resulting in concentrations ranging from 0.3125 to 40 mg/mL. Bacterial suspensions, adjusted to 10⁶ CFU/mL from overnight cultures, were also prepared in MHB. Following the addition of 100 μL bacterial inoculum with 100 μL EO samples, the plates were shaken and then incubated at 37 °C for 24 h. The final sample concentrations ranged from 0.1562 to 20.0 mg/mL. The 10th well, regarded as a negative control, contained 200 μL of MHB and 5% DMSO without bacterial suspension, while the 12th well, with 100 μL of MHB and 5% DMSO along with 100 μL bacterial suspension, served as a positive control. The 9th and 11th wells separated each assay. Three replications were conducted for each EO dilution.

### 3.5. Statistical Analysis

The results were expressed as means ± standard deviations (SD) derived from three measurements per test. For each chemical family (esters, hydrocarbon monoterpenes, hydrocarbon sesquiterpenes, oxygenated monoterpenes, oxygenated sesquiterpenes, and phenylpropenes) found in DCEO and FVEO, the variation of the chemical component content between the two plant EOs was tested using paired *t*-tests at α = 0.05.

The similarity in chemical composition among the two plant species EOs was analyzed using both qualitative similarity indices (Jaccard index and Sørensen index) computed based on the presence/absence of the chemical components and quantitative indices (Chao’s abundance-based Jaccard index, Chao’s abundance-based Sørensen index, Morisita–Horn index, and Bray–Curtis index), which were determined based on component percent data. 

Using the package ecotox [76] built under the statistical R program version 4.4.0 [77], the 24 h mortality data for *C. pipiens* larvae were subjected to log probit analysis, using regression between log EO concentration and probit values to determine concentrations leading to 50%, 95%, and 99% mortality (LC_50_, LC_95_, LC_99_) with 95% confidence intervals. The variation in values of IZDs among DCEO, FVEO, and GMN10 was tested using one-way ANOVA followed by Tukey’s post hoc test (HSD).

## 4. Conclusions

The current study presents a comprehensive and comparative analysis of the chemical composition, as well as the larvicidal and antibacterial effects of EOs from the aerial parts of two wild Apiaceae plants, *D. carota* and *F. vulgare*. GC/MS analysis showed that DCEO was characterized by its oxygenated monoterpenes, and FVEO was abundant in monoterpenes and phenylpropenes. Both oils demonstrated interesting larvicidal activity, suggesting their potential as alternative bioinsecticides to reduce the transmission of *C. pipiens*, the most prevalent mosquito species in Algeria. The production of bioinsecticides for managing mosquito larval populations is strongly advocated, given the toxicity of many synthetic insecticides currently in use and the rising levels of their resistance. Future research could explore the employment of these oils in various formulations, such as larvicidal sprays or granules, to develop innovative biopesticides and new sustainable strategies for mosquito control. Furthermore, both EOs exhibited weak to moderate antibacterial activity against gram-negative and gram-positive bacteria, respectively. This approach underscores the importance of synergistic interactions between EO components, as even minor compounds can impact overall effectiveness. It is well known that combining substances can boost efficacy. Future research may focus on using DCEO and FVEO either in combination with each other or with other bioactive compounds, nanoparticles, or antibiotics.

## Figures and Tables

**Table 1:** GC/MS profiles of DCEO and FVEO.

N°	Compound Name	Identification	Chemical Family	KI Lit ^1^	DCEO			FVEO		
					Rt ^2^ (min)	KI Exp ^3^	Area ^4^ (%)	Rt ^2^ (min)	KI Exp ^3^	Area ^4^ (%)
1	α-Thujene	S, MS, KI	Hydrocarbon monoterpene	929	7.617	932	0.033	7.608	931	0.176
2	α-Pinene	S, MS, KI	Hydrocarbon monoterpene	937	7.850	939	**5.437**	7.842	938	**7.360**
3	Camphene	S, MS, KI	Hydrocarbon monoterpene	952	8.375	954	0.324	8.367	954	0.296
4	Sabinene	S, MS, KI	Hydrocarbon monoterpene	974	9.292	977	0.687	9.283	977	0.347
5	β-Pinene	S, MS, KI	Hydrocarbon monoterpene	979	9.408	980	0.658	9.400	980	0.825
6	β-Myrcene	S, MS, KI	Hydrocarbon monoterpene	991	9.958	993	**2.063**	9.950	993	**2.884**
7	α-Phellandrene	S, MS, KI	Hydrocarbon monoterpene	1005	10.483	1006	0.601	10.483	1006	**17.958**
8	3-Carene	S, MS, KI	Hydrocarbon monoterpene	1011	10.717	1012	0.150	10.708	1012	0.476
9	α-Terpinene	S, MS, KI	Hydrocarbon monoterpene	1017	-	-	-	10.975	1019	0.076
10	*p*-Cymene	S, MS, KI	Hydrocarbon monoterpene	1025	11.300	1028	0.453	11.292	1028	**4.879**
11	Limonene	S, MS, KI	Hydrocarbon monoterpene	1030	11.475	1032	**4.229**	-	-	-
12	β-Phellandrene	S, MS, KI	Hydrocarbon monoterpene	1031	-	-	-	11.475	1032	**9.144**
13	*cis-*β-Ocimene	MS, KI	Hydrocarbon monoterpene	1038	-	-	-	11.867	1042	0.397
14	*trans*-β-Ocimene	MS, KI	Hydrocarbon monoterpene	1049	11.883	1043	0.028	12.308	1053	0.066
15	α-Ocimene	MS, KI	Hydrocarbon monoterpene	1047	12.325	1053	0.062	-	-	-
16	γ-Terpinene	S, MS, KI	Hydrocarbon monoterpene	1060	12.775	1063	0.037	12.758	1063	0.8
17	Fenchone	S, MS, KI	Oxygenated monoterpene	1096	14.025	1090	0.539	14.025	1090	**20.202**
18	α-Pinene oxide	MS, KI	Oxygenated monoterpene	1095	14.475	1099	0.025	-	-	-
19	Linalool	S, MS, KI	Oxygenated monoterpene	1099	14.558	1100	0.776	14.567	1101	0.075
20	*cis-p*-Mentha-2,8-dien-1-ol	MS, KI	Oxygenated monoterpene	1102	14.833	1107	0.213	-	-	-
21	Fenchol	S, MS, KI	Oxygenated monoterpene	1113	15.158	1116	0.017	15.150	1115	0.047
22	*Exo*-Fenchol	MS, KI	Oxygenated monoterpene	1116	-	-	-	15.325	1120	0.040
23	*cis*-2-Menthenol	MS, KI	Oxygenated monoterpene	1122	15.508	1124	0.104	15.517	1124	0.072
24	Pinocarveol	S, MS, KI	Oxygenated monoterpene	1139	16.292	1142	0.078	-	-	-
25	*trans*-2-Menthenol	MS, KI	Oxygenated monoterpene	1140	-	-	-	16.325	1143	0.042
26	Camphor	S, MS, KI	Oxygenated monoterpene	1145	-	-	-	16.525	1148	0.272
27	*trans*-Verbenol	MS, KI	Oxygenated monoterpene	1144	16.567	1148	0.261	-	-	-
28	Pinocarvone	MS, KI	Oxygenated monoterpene	1164	17.383	1166	0.02	-	-	-
29	*endo*-Borneol	MS, KI	Oxygenated monoterpene	1167	17.517	1169	0.046	17.642	1172	0.054
30	4-terpineol	S, MS, KI	Oxygenated monoterpene	1182	-	-	-	18.050	1180	0.208
31	Terpinen-4-ol	S, MS, KI	Oxygenated monoterpene	1177	18.058	1180	0.386	-	-	-
32	Dihydrocarvone	S, MS, KI	Oxygenated monoterpene	1179	-	-	-	18.342	1186	0.009
33	α-Terpineol	S, MS, KI	Oxygenated monoterpene	1189	18.675	1192	0.101	18.658	1192	0.059
34	Myrtenal	S, MS, KI	Oxygenated monoterpene	1193	18.925	1197	0.063	-	-	-
35	Estragole	S, MS, KI	Phenylpropene	1196	19.033	1199	0.091	19.050	1200	**24.928**
36	*cis*-Piperitol	S, MS, KI	Oxygenated monoterpene	1203	-	-	-	19.175	1201	0.222
37	*trans*-Piperitol	S, MS, KI	Oxygenated monoterpene	1208	-	-	-	19.458	1210	0.053
38	Verbenone	MS, KI	Oxygenated monoterpene	1205	19.508	1211	0.148	-	-	-
39	*cis*-Carveol	MS, KI	Oxygenated monoterpene	1229	19.967	1222	0.068	-	-	-
40	Fenchyl acetate	S, MS, KI	Oxygenated monoterpene	1223	-	-	-	19.975	1222	0.158
41	*exo*-2-Hydroxycineole	MS, KI	Oxygenated monoterpene	1224	-	-	-	20.15	1226	0.049
42	Isogeraniol	MS, KI	Oxygenated monoterpene	1240	-	-	-	20.592	1237	0.399
43	*cis*-Sabinol	MS, KI	Oxygenated monoterpene	1243	-	-	-	20.775	1241	0.019
44	Pulegone	S, MS, KI	Oxygenated monoterpene	1237	20.867	1243	0.136	-	-	-
45	Carvone	S, MS, KI	Oxygenated monoterpene	1242	21.075	1248	0.068	-	-	-
46	Piperitone oxide	MS, KI	Oxygenated monoterpene	1256	-	-	-	21.442	1256	0.486
47	Geraniol	S, MS, KI	Oxygenated monoterpene	1255	21.567	1259	0.277	-	-	-
48	Geranial	S, MS, KI	Oxygenated monoterpene	1270	22.292	1274	0.119	-	-	-
49	Citronellyl formate	MS, KI	Oxygenated monoterpene	1276	22.792	1285	0.020	-	-	-
50	Anethole	S, MS, KI	Phenylpropene	1283	-	-	-	22.933	1288	**4.481**
51	Bornyl acetate	MS, KI	Oxygenated monoterpene	1285	22.942	1288	0.495	-	-	-
52	Carvacrol	S, MS, KI	Oxygenated monoterpene	1299	-	-	-	23.717	1304	0.033
53	Myrtenyl acetate	MS, KI	Oxygenated monoterpene	1327	24.700	1328	0.065	25.008	1336	0.062
54	Pinanediol	MS, KI	Oxygenated monoterpene	1316	-	-	-	24.283	1318	0.771
55	Limonene glycol	MS, KI	Oxygenated monoterpene	1321	-	-	-	24.792	1331	0.026
56	δ-Elemene	MS, KI	Hydrocarbon sesquiterpene	1338	25.225	1341	0.091	-	-	-
57	Longipinene	MS, KI	Hydrocarbon sesquiterpene	1353	25.767	1354	0.309	-	-	-
58	Longicyclene	MS, KI	Hydrocarbon sesquiterpene	1374	26.167	1363	0.029	-	-	-
59	Nerol acetate	MS, KI	Oxygenated monoterpene	1364	26.392	1368	0.067	-	-	-
60	Copaene	MS, KI	Hydrocarbon sesquiterpene	1376	26.883	1379	0.180	26.875	1379	0.042
61	Geranyl acetate	MS, KI	Oxygenated monoterpene	1382	27.275	1387	**50.074**	-	-	-
62	β-Cubebene	MS, KI	Hydrocarbon sesquiterpene	1389	27.500	1392	0.047	-	-	-
63	β-Elemene	MS, KI	Hydrocarbon sesquiterpene	1391	27.583	1394	0.022	-	-	-
64	2,3-epoxy-Geranyl acetate	MS, KI	Oxygenated monoterpene	1393	-	-	-	27.608	1394	0.202
65	β-Longipinene	MS, KI	Hydrocarbon sesquiterpene	1403	27.892	1401	0.031	-	-	-
66	Longifolene	MS, KI	Hydrocarbon sesquiterpene	1405	28.142	1407	0.158	-	-	-
67	α-Cedrene	MS, KI	Hydrocarbon sesquiterpene	1411	28.433	1415	0.056	-	-	-
68	Caryophyllene	S, MS, KI	Hydrocarbon sesquiterpene	1419	28.75	1423	0.294	-	-	-
69	*trans*-α-Bergamotene	S, MS, KI	Hydrocarbon sesquiterpene	1435	29.417	1439	0.120	-	-	-
70	*cis*-β-Farnesene	MS, KI	Hydrocarbon sesquiterpene	1444	29.717	1447	0.187	-	-	-
71	Humulene	S, MS, KI	Hydrocarbon sesquiterpene	1454	30.15	1457	0.073	-	-	-
72	*trans*-β-Farnesene	S, MS, KI	Hydrocarbon sesquiterpene	1457	30.275	1460	0.180	-	-	-
73	Germacrene D	S, MS, KI	Hydrocarbon sesquiterpene	1481	31.100	1480	0.073	31.275	1484	0.094
74	Methylvanillin	MS, KI	Aromatic aldehyde	1495	31.283	1484	0.500	-	-	-
75	6-Hydroxy-3,7-dimethyl-2,7-octadienyl acetate(E)	MS, KI	Ester	1496	31.592	1491	0.535	-	-	-
76	Methylisoeugenol	MS, KI	Phenylpropene	1492	31.950	1499	**2.206**	-	-	-
77	β-Himachalene	MS, KI	Hydrocarbon sesquiterpene	1500	32.083	1503	0.130	-	-	-
78	β-Bisabolene	MS, KI	Hydrocarbon sesquiterpene	1509	32.392	1511	**2.401**	-	-	-
79	Isolongifolan-8-ol	MS, KI	Oxygenated sesquiterpene	1523	32.733	1520	0.033	-	-	-
80	δ-Cadinene	MS, KI	Hydrocarbon sesquiterpene	1524	32.983	1527	0.137	32.975	1527	0.048
81	*ar*-Himachalene	MS, KI	Hydrocarbon sesquiterpene	1542	33.550	1542	0.168	-	-	-
82	*cis*-Sesquisabinene hydrate	MS, KI	Oxygenated sesquiterpene	1543	33.733	1546	0.145	-	-	-
83	Myrtenyl isovalerate	MS, KI	Oxygenated sesquiterpene	1552	33.983	1553	0.027	-	-	-
84	Elemicin	MS, KI	Phenylpropene	1554	34.317	1561	**10.767**	-	-	-
85	Nerolidol	S, MS, KI	Oxygenated sesquiterpene	1564	34.550	1567	0.071	-	-	-
86	Spathulenol	MS, KI	Oxygenated sesquiterpene	1576	35.125	1581	0.059	-	-	-
87	Caryophyllene oxide	S, MS, KI	Oxygenated sesquiterpene	1581	35.250	1586	0.032	-	-	-
88	Viridiflorol	S, MS, KI	Oxygenated sesquiterpene	1591	35.675	1595	0.083	-	-	-
89	Longiborneol	MS, KI	Oxygenated sesquiterpene	1592	35.875	1600	0.028	-	-	-
90	Humulene epoxide 2	MS, KI	Oxygenated sesquiterpene	1606	36.342	1613	0.057	-	-	-
91	β-Himachalene oxide	MS, KI	Oxygenated sesquiterpene	1615	36.467	1616	0.063	-	-	-
92	Farnesene epoxide	MS, KI	Oxygenated sesquiterpene	1624	36.775	1625	0.040	-	-	-
93	Isospathulenol	MS, KI	Oxygenated sesquiterpene	1638	37.075	1633	0.094	-	-	-
94	*epi*-Cubenol	MS, KI	Oxygenated sesquiterpene	1627	37.200	1636	0.095	-	-	-
95	Himachalol	MS, KI	Oxygenated sesquiterpene	1647	37.683	1649	**5.919**	-	-	-
96	Isoelemicin	MS, KI	Phenylpropene	1654	37.908	1655	0.058	-	-	-
97	α-Cadinol	MS, KI	Oxygenated sesquiterpene	1653	38.025	1659	0.028	-	-	-
98	*cis*-10-Hydroxycalamene	MS, KI	Oxygenated sesquiterpene	1666	38.167	1662	0.037	-	-	-
99	Triethyl citrate	MS, KI	Ester	1658	38.325	1666	0.146	-	-	-
100	Asarone	S, MS, KI	Phenylpropene	1678	39.008	1684	0.493	-	-	-
101	α-Bisabolol	S, MS, KI	Oxygenated sesquiterpene	1684	39.175	1689	0.306	-	-	-
102	Juniper camphor	MS, KI	Oxygenated sesquiterpene	1692	39.567	1699	0.220	-	-	-
103	β-Santalol	MS, KI	Oxygenated sesquiterpene	1715	40.033	1712	0.132	-	-	-
104	Acoramone	MS, KI	Oxygenated sesquiterpene	1751	41.442	1753	0.090	-	-	-
Number of compounds					84			42		
Hydrocarbon monoterpenes					14.762			45.710		
Oxygenated monoterpenes					54.166			23.534		
Hydrocarbon sesquiterpenes					4.686			0.184		
Oxygenated sesquiterpenes					7.559			-		
Others *					14.796			29.409		
Sum (%)					95.97			98.84		
Yield (%)					0.80			0.85		

DCEO: *Daucus carota* essential oil. FVEO: *Foeniculum vulgare* essential oil. S: standard. MS: comparison of mass spectra. KI: comparison of experimental and literature Kovats indices. ^1^ Kovats retention indices according to the NIST20 database. ^2^ Retention Times. ^3^ Experimental Kovats retention indices calculated against n-alkanes. ^4^ Area (%) according to TIC-MS chromatogram. Areas (%) ≥ 1 are presented in bold. ‘-’: Absent. * Phenylpropene, ester, and aromatic aldehyde.

**Table 2:** Mosquito larvicidal potential of DCEO, FVEO, and the insecticide “Deltamethrin” against *Culex pipiens* larvae at 24 h post-treatment.

Treatment	Conc. (ppm)	Mortality (%) ± SE	LC_50_ (ppm) ± SE (LCL–UCL)	LC_95_ (ppm) ± SE (LCL–UCL)	LC_99_ (ppm) ± SE (LCL–UCL)	Slope ± SE	*χ* ^2^
DCEO	6.25	0.00 ± 0.00	30.6 ± 1.06 (27.1–34.3)	86.5 ± 1.1 (73–108)	133 ± 1.14 (107–178)	3.65 ± 0.307	11.4
	12.5	13.33 ± 2.57					
	25	28.33 ± 6.01					
	50	80.00 ± 2.89					
	75	91.67 ± 1.67					
	100	96.67 ± 3.33					
FVEO	6.25	0.00 ± 0.00	34.7 ± 1.06 (30.6–39)	106 ± 1.11 (88.4–135)	169 ± 1.15 (133–233)	3.38 ± 0.29	13.2
	12.5	11.67 ± 1.67					
	25	28.33 ± 1.67					
	50	63.33 ± 7.26					
	75	85.00 ± 2.89					
	100	100.00 ± 0.00					
Deltamethrin	0.1	0.00 ± 0.00	0.367 ± 1.03 (0.345–0.388)	0.626 ± 1.06 (0.571–0.712)	0.782 ± 1.08 (0.691–0.931)	7.08 ± 0.683	14.8
	0.2	6.67 ± 1.67					
	0.3	26.67 ± 3.33					
	0.4	46.67 ± 3.33					
	0.5	83.33 ± 3.33					
	0.6	100.00 ± 0.00					

DCEO: *Daucus carota* essential oil. FVEO: *Foeniculum vulgare* essential oil. Conc.: concentration. ppm: parts per million. LC_50_: Concentration for 50% mortality, with 95% confidence limit. LC_95_: Concentration for 95% mortality, with 95% confidence limit. LC_99_: Concentration for 99% mortality, with 95% confidence limit. LCL: Lower Confidence Limit. UCL: Upper Confidence Limit. Slope: Slope of the concentration mortality regression line. SE: standard error; *χ*^2^: Chi-square value.

**Table 3:** Inhibition zone diameters and minimum inhibitory concentration values of DCEO and FVEO.

Bacterial Species	Values	EO Source		GMN10	ANOVA	
		DCEO	FVEO		*F*-Statistics	*p*-Value
*Escherichia coli* ATCC 25922	IZD (mm)	7.0 ± 0.0 ^A^	9.3 ± 0.6 ^B^	30.0 ± 0.0 ^C^	4327	<0.001
	MIC (mg/mL)	ND	ND	ND	—	—
*Klebsiella pneumonia* ATCC 700603	IZD (mm)	8.0 ± 0.0 ^A^	9.3 ± 0.6 ^B^	25.0 ± 0.0 ^C^	2413	<0.001
	MIC (mg/mL)	ND	ND	ND	—	—
*Staphylococcus aureus* ATCC 25923	IZD (mm)	24.0 ± 1.7 ^C^	10.7 ± 0.6 ^A^	20.3 ± 0.6 ^B^	116.5	<0.001
	MIC (mg/mL)	10.0 ± 0.0	>20.0	ND	—	—
*Staphylococcus aureus* MRSA ATCC 43300	IZD (mm)	18.7 ± 1.2 ^B^	10.3 ± 0.6 ^A^	25.0 ± 0.0 ^C^	292.2	<0.001
	MIC (mg/mL)	20.0 ± 0.0	>20.0	ND	—	—

IZD: inhibition zone diameter. MIC: minimum inhibitory concentration. DCEO: *Daucus carota* essential oil. FVEO: *Foeniculum vulgare* essential oil. GMN10: Gentamicin 10 µg. ND: not determined. *F*-statistics and *p*-value are results of one-way ANOVA. Different superscript letters associated with means ± SD inhibition zone diameter indicate significant difference following HSD Tukey test at *p* < 0.05.

## Data Availability

The datasets used and/or analyzed during the current study are available from the corresponding author on reasonable request.
